# Nucleosome Dancing at the Tempo of Histone Tail Acetylation

**DOI:** 10.3390/genes6030607

**Published:** 2015-07-15

**Authors:** Angélique Galvani, Christophe Thiriet

**Affiliations:** UMR CNRS 6286 UFIP, Université de Nantes, Epigénétique: Proliferation et Différenciation, 2 rue de Houssinière, 44322 Nantes Cedex 03, France; E-Mail: Angelique.Galvani@univ-nantes.fr

**Keywords:** histone acetylation, transcription, chromatin dynamics

## Abstract

The impact of histone acetylation on transcription was revealed over 50 years ago by Allfrey and colleagues. However, it took decades for an understanding of the fine mechanism by which this posttranslational modification affects chromatin structure and promotes transcription. Here, we review breakthroughs linking histone tail acetylation, histone dynamics, and transcription. We also discuss the histone exchange during transcription and highlight the important function of a pool of non-chromatinized histones in chromatin dynamics.

## 1. Introduction

In eukaryotic cells, the genome is packaged into the nucleus in chromatin. The basic subunit of chromatin is the nucleosome, which is composed of 147 base pairs of DNA wrapped around a histone octamer comprising two copies each of H2A, H2B, H3, and H4 [1]. Even though chromatin can be represented as beads on a string, different condensation states co-exist in the nucleus, thereby compartmentalizing the genetic information into euchromatin and heterochromatin. The different levels of compaction of chromatin have been associated with non-histone proteins and with posttranslational modifications of histones [2]. As an example, the condensation of chromatin in heterochromatin has been linked with the methylation of the histone H3 on lysine 9, which has been shown to promote the association of the heterochromatin protein 1 (HP1) [3]. The fundamental cellular processes of replication, transcription, and repair must therefore function within an initially condensed substrate. To achieve access to DNA that is complexed in chromatin, the cell has evolved mechanisms that alter chromatin structures and promote access to the genetic information. Examples of this include the posttranslational modifications of histones, the utilization of ATP-dependent chromatin remodelers, and the replacement of canonical histones by histone variants.

While it has been long known that histones can be posttranslationally modified, the past decades have been particularly productive in this field. This breakthrough has at least partially been due to the improvement of methods to detect and identify the presence of modified residues, methods such as mass spectroscopy and the synthesis of modified peptides that allow the generation of specific antibodies. To date, several hundreds of histone modifications have been reported. This implies that posttranslational modifications may be used sequentially or in combination to achieve distinct functional consequences, hence the proposal that a histone code exists [4,5]. Though the existence of an actual histone code is controversial and much debated, it is obvious that the number of histone posttranslational modifications exceeds the variability of chromatin activities, which raises the question of the meaning of this diversity. As well as the large variety of posttranslational modifications, it has been shown that the addition of chemical groups to histones improves their interaction with specific structural motifs of other chromatin proteins, and thus has a role in histone recruitment. Hence, it has been proposed that “writers” would modify the histones, “readers” would interact with the modified histones, and “erasers” would remove the histone modification.

It is generally believed that the alteration of chromatin structure should be a primary event to access genetic information. Genetic screenings in yeast have identified the SWI/SNF proteins as global regulators of transcription. Further characterization of the SWI/SNF proteins revealed that they resided within a single large complex of ~2 mega Daltons and presented ATPase activity [6]. *In vitro* analyses of the SWI/SNF complex have shown that the ATPase activity is required for disrupting histone–DNA interactions. Interestingly, the isolation of complexes presenting ATP-dependent chromatin remodeling activities in other organisms, including *Drosophila* and human, has reinforced the idea that these complexes have important functions [7]. Members of the family of the ATP-dependent chromatin remodelers have conserved features, including a requirement for ATPase activity to disrupt histone-DNA interactions and the formation of multi-subunit complexes [8]. Further characterizations of the function of the ATP-dependent chromatin remodelers have shown that these complexes are associated with cellular processes that require the relief of chromatin-mediated repression, such as transcription of inducible genes, or transcription leading to cell cycle progression, cell differentiation, and repair [9,10,11,12]. Even though the ATP-dependent remodeler complexes can exhibit chromatin process specialization, depending upon the specific subunits composing the complexes, the mechanism of remodeling is conserved. In general, remodeling disrupts histone-DNA contacts (as revealed by the alteration of the DNase I nucleosome footprint pattern), and can result in histone octamer sliding along the DNA [13].

Genetic analyses have demonstrated that the compaction of the genetic material into nucleosomes is required for the cell survival. However, histones composing nucleosomes are not identical in sequence throughout the entire genome. In addition to sequence divergence, histone variants have been shown to be expressed throughout the cell cycle, in contrast to the “canonical” histones that are expressed only during S-phase [14,15]. Except for histone H4, all classes of histones have histone variants. It has been shown that histone variants can be used as specific markers of the state of chromatin, and can provide a map of specialized genome regions. To support this, it has been demonstrated that the specific phosphorylation of the variant H2A-X colocalizes with double-stranded DNA breaks, and triggers the repair process [12,16]. Histone variants such as H3.3 and H2A-Z have also been associated with transcription [17]. Interestingly, reconstitution of the nucleosome with these two variants revealed weaker stability than canonical histone-containing nucleosomes [18].

The mechanisms for promoting access to genetic information have been related to chromatin processes. However, the absence of an exclusive mechanism implies inter-dependence between the different factors for regulating genetic activities. The interplay between distinct mechanisms requires perfect coordination, in which histone modifications have a central role. Indeed, histone modifications can alter chromatin structure by changing the global charge and/or the structure of the histone, while also recruiting factors (usually referred to as “readers”) that present specific domains that interact with those modifications.

In the present paper, we review the different alterations of chromatin needed for transcriptional activation. Although the focus is primarily on the mechanisms leading to polymerase II transcription, it is clear that our knowledge of the removal of nucleosomal hindrance has also been improved using other model processes. Gene transcription is a very well-regulated process that comprises control elements within the promoter region and elongation by RNA polymerase within the coding region. Even though histone tail acetylation is clearly not the unique modification associated with transcription, it has historically been the first identified, and therefore the focus of numerous studies over several decades. Thus, we propose to spotlight the function of acetylation of the histone tail domains in chromatin dynamics in conjunction with transcription.

## 2. The Nucleosome: An Impediment for Transcription Factor Binding

Understanding the mechanisms by which transcription occurs in the presence of the nucleosome has been the focus of a large number of studies. Biochemical analyses of the nucleosome have enabled us to determine the extent of inhibition that histones can generate within the nucleosomal structure. Several DNA fragments have been used to examine the binding of transcription factors to the nucleosome. Among the most commonly studied are (1) the DNA fragment from *Xenopus* comprising the gene encoding for 5S rRNA and the transcription factor TFIIIA [19]; (2) the GAL4 binding site-containing DNA fragment; and (3) the NF-κB binding site-containing DNA fragment [20,21]. All these model systems were reconstituted into a nucleosome for investigating the binding of the transcription factor. The results clearly demonstrated that the recognition site of transcription factors is occluded, at least partially, when interacting with a core histone octamer. However, Widom and colleagues have shown that DNA within the nucleosome is flexible, as demonstrated by the analyses of restriction site accessibility to enzymatic digestion [22,23,24]. These results suggested that even though the histone-DNA contacts are continuously disrupted and reformed, these dynamics do not support transcription factor binding. The reason for the discrepancy between restriction enzyme recognition and transcription factor binding may lie in the size difference between the recognition sites of the restriction enzymes and the transcription factors; or in the fact that transcription factors are more sensitive to histone steric hindrance than are restriction enzymes.

## 3. Relieving the Nucleosome Barrier by Histone Acetylation

Proteolysis experiments using trypsin digestion on chromatin have revealed that histones are composed of two regions, the tail domains that can be removed by the trypsin and the fold domain that is protected from digestion and involved in nucleosome structure. The removal of the histone tail domains within the nucleosome has revealed the abolishment of the inhibition of TFIIIA binding onto its recognition site [25]. Furthermore, the deletion of specific histone tail domains within the histone octamer exhibited binding of the transcription factor TFIIIA to its recognition site when tailless H3 and H4 were reconstituted into nucleosomes [26]. It was thus proposed that the tail domains of the (H3/H4)_2_ tetramer presented the major impediment, and occluded transcription factors binding. Even though the removal of the histone tail domains pinpointed an important role of these domains in the accessibility to DNA sequences, it did not provide information on the mechanism that the cell has developed for relieving the histone tail hindrance. Interestingly, a 5S gene-containing DNA fragment reconstituted with hyper-acetylated histones recapitulated TFIIIA binding and provided a mechanistic function of the histone tail acetylation in transcription [25]. These results were consistent with the analyses of the binding of acetylated H4 peptides and unacetylated H4 peptides to DNA. Indeed, thermal denaturation experiments have exhibited a strongly reduced affinity of the fully acetylated H4 peptides compared to the unacetylated version of the same peptide sequence [27]. It has been proposed that the acetylation-dependent decrease of affinity of the tail domains was due to the neutralization of the positive charge of the lysine residues. Undoubtedly the addition of the acetyl group to the lysine alters the charge; however, it has been also reported that the acetylation increased the α-helical content of the histone tail domains [28]. This suggests that histone acetylation affected the global charge and induced some structures within tail domains that allowed access to DNA.

## 4. Relaxing the Chromatin Structure with Nucleosome Acetylation

Although *in vitro* experiments have provided a demonstration of the positive effect of histone acetylation on recognition site access in nucleosomal context for transcription factors, this required a high level of acetylation, especially of H4, for significantly enhancing transcription factor binding. It is unclear whether such a high level of acetylation is necessary *in vivo*. Indeed, the mononucleosome model system is certainly significant and well suited for studying the extent of histone impediment within the nucleosome; however, it might not reflect the influence of neighboring nucleosomes and the folding of oligonucleosome arrays. For instance, it has been shown that cells treated with sodium butyrate, which induced chromatin hyperacetylation, exhibit a greater accessibility to nuclease than untreated cells [29]. Nonetheless, a mononucleosome reconstituted with acetylated histone revealed similar footprinting to a non-acetylated nucleosome [25]. Hence, this showed that histone acetylation affects the chromatin structure by limiting the stacking of nucleosomes rather than affecting the nucleosomal structure itself. In support of this, precipitation experiments of nucleosomal arrays by MgCl_2_ have demonstrated that oligonucleosome arrays reconstituted with acetylated histones folded less efficiently than unmodified histones. This suggests that acetylation reduces inter-nucleosomal interactions and affects the stacking of the nucleosomes, and thus contributes to the chromatin opening required for transcription [30].

## 5. Painting Chromatin with Histone Tail Acetylation

Earlier studies had shown that heat shock gene activation in *Drosophila* was concomitant with alteration of chromatin structure at the promoter region [31,32]. Using the yeast model system, the Hörz group has characterized the different steps of activation of PHO genes upon inorganic phosphate starvation [33]. Even though the activation of these genes exhibited an opening of chromatin that resulted in the loss of nucleosomes within the promoter region, these alterations of chromatin involved histone acetylation and chromatin remodeling [34,35]. Indeed, in the absence of chromatin remodeling by the Swi2-containing chromatin remodeler, histone acetylation proceeded but transcription was not activated [36]. With these experiments, the authors demonstrated that histone acetylation is not sufficient to achieve transcription, but coordinates other processes such as nucleosome remodeling. Structural analyses of remodeler complexes have revealed the presence of a specific motif (named the bromodomain) of about 110 amino acids that has been identified for its ability to bind specifically to acetyl-lysine [37,38]. Interestingly, the bromodomain is conserved throughout evolution, and is present in a number of transcription-related factors, such as Gcn5, Snf2, and Sth1 [39]. However, in contrast to the expectation, pull-down experiments have shown that the bromodomain of Gcn5 can interact specifically with the histone tail domains even in the absence of acetylated lysine [40]. Furthermore, NMR analyses of bromodomains have revealed that the acetyl-lysine recognition is specific but the dissociation constant Kd is weak, in the μM–mM range [38]. These data suggested that histone tail acetylation recruits factors via their bromodomains.

One of the major breakthroughs in understanding of role of histone acetylation *in vivo* has been the development of specific antibodies to probe specific acetyl-lysines of histones. Using such probes to examine the histone acetylation within polytene chromosome, Turner and colleagues have shown the presence of specific patterns of acetylation of H4 within distinct chromosomal regions that correlate with different transcriptional activities [41]. These primary observations were then generalized to other eukaryotes with the development of the chromatin immunoprecipitation technique (ChIP) and the analyses of locus models. These results supported the idea that histone acetylation might provide a specific signal, and be used as a platform for factors involved in transcription.

## 6. Histone Dynamics in Transcription

Using an elegant *in vitro* approach to reconstitute RNA polymerase II elongation, Studitsky and collaborators have shown that RNA polymerase II can progress through a nucleosome by inducing the eviction of one H2A/H2B dimer [42]. With a similar experimental approach it has been shown that the histone chaperone FACT facilitates this process [43]. The need for a histone chaperone like FACT in the histone dimer eviction is unsurprising as free histones present high affinity for any nucleic acid, and must be guarded by chaperones to prevent the formation of unspecific nucleoprotein complexes that might be toxic to the cell. Therefore, any histone dynamics-related mechanism implies the coordinated formation of histone-histone chaperone complexes to neutralize the negative charge of histones, and to facilitate the recycling of histones. To date, a large body of literature has focused on the identification of histone chaperones and their specificity to histones. Nonetheless, the level of histone acetylation was undetermined in these experiments of transcription through a nucleosome, and thus it is unclear whether histone tail acetylation is critical for the processivity of RNA polymerase II. Interestingly, *in vivo* analyses of the core histone dynamics at transcriptionally active loci using the *Physarum* model system revealed that H2A/H2B dimers have a greater rate of turnover in RNA polymerase II coding regions [44].

Beyond the correlation between the histone acetylation and transcription, the works of Ahmad and Henikoff have revealed in *Drosophila* that active chromatin regions are enriched in the H3.3 histone variant, which has only a few amino acid residue differences relative to canonical H3 [45,46]. Although these observations were particularly pronounced in rDNA loci, which are transcribed by RNA polymerase I, the exchange of canonical H3 by the H3.3 variant was also verified by genome-scale profiling in transcriptionally active chromatin [47]. These results demonstrated that the exchange of H3 with H3.3 is correlated with the general transcription process and is not restricted to RNA polymerase I. Nonetheless, it is likely that the greatest rate of exchange of the canonical H3 in the polymerase I transcribed region is more related to the velocity of the transcription of these specific regions, which encode ribosomal RNAs [48]. To reinforce a putative role of the H3.3 variant in transcription, the analyses of the posttranslational modifications revealed that H3.3 is enriched in transcription associated modifications (K4 and K79 methyl; K9, K14, K18, and K23 acetyl) and deficient in dimethyl lysine-9, which preferentially marks heterochromatin [49]. Unexpectedly, knock-out experiments of the H3.3 gene in *Tetrahymena* and in human cells did not exhibit a phenotype reflecting failures in transcription activities [50,51,52]. These results showed that H3.3 is not essential for transcription, although the accumulation of this histone variant at the transcribed region is demonstrated in different cell types. Importantly, genetic analyses of the function of the H3.3 histone variant in *Tetrahymena* highlighted that, rather than its particular primary sequence, the constitutive expression of the replacement histone variant H3.3 was the important feature [50]. In the budding yeast, *Saccharomyces cerevisiae*, the only H3 available resembles H3.3 and is deposited into chromatin through both replication-coupled and replication-independent chromatin assembly pathways [53]. By tracing inducible forms of epitope-tagged histones in yeast, Strubin and collaborators have investigated the nucleosome dynamics in G1 phase of the cell cycle. The results revealed that a tagged version of H3 accumulated in active loci, while tagged H2B was recovered in both active and inactive loci [54]. In this report, the authors have also examined histone exchange with N-terminally truncated histones, and found identical patterns of dynamics compared to full-length histones. Hence, they concluded that replication-independent incorporation of the histones occurred independently of the histone tail domains, and is evidently unlinked to histone tail modifications. However, it was unclear whether the rate of histone exchange was affected by the absence of the tail domains and therefore if the removal of the histone tails is a pertinent mimic of histone tail modifications.

## 7. The Dynamic Equilibrium of Histone Acetylation

Analyses of histone acetylation over large genome regions have revealed that the modification is not restricted to the promoter but also extended within the coding region. The analyses of histone modification over the β-globin domain have illustrated that the opening of this chromatin region in conjunction with histone acetylation was not limited to the promoter [55,56,57,58]. While the acetylation at the promoter region has been proposed to act as a platform that would recruit bromodomain-containing transcription factors, the processive activity of RNA polymerase along the coding region did not require such recruitment [34]. Nonetheless, the *in vitro* analyses of oligonucleosome models revealed that histone acetylation affected the formation of higher order structures, and the wrapping of DNA within a nucleosome [25,30,59]. These effects of histone acetylation supported, therefore, the requirement of these modifications to enhance the passage of RNA polymerase through nucleosomes.

In *Physarum*, the state of histone acetylation has been investigated throughout the cell cycle using the synchronous population of nuclei contained within a single cell [60,61,62,63]. Experiments examining the incorporation of tritiated acetate into histones have revealed that the pattern of acetylation is correlated with the cell cycle stage, at least in this model organism [62]. Indeed, while all S-phase core histones exhibited acetylated forms, during G2-phase of the cell cycle only H3 and H4 were predominantly acetylated [63]. Experiments using radioactive precursors, such as tritiated acetate (for acetylation), tritiated SAM (for methylation), and radioactive γATP (for phosphorylation), did not readily improve the understanding of specific histone modification patterns in correlation with chromatin activities; however, they have provided information on the duration of the modifications within the histones. This contrasts with the usage of modification specific antibodies, which can specifically recognize modified histone residues, and therefore discriminate between the presence and absence of the modification. Hence, radioactive precursors have demonstrated that the half-life of the acetyl group of the acetylated histones is short, in the range of a few minutes, and depends upon the cell type and organism. The detailed analyses of the half-life of the acetyl group in *Chlamydomonas reinhardii* showed that in the absence of translation of new histones, the half-life of the acetyl group within core histones was less than 5 min [64]; the persistence of histone acetylation corresponding to the steady state was significantly longer. The divergence between the half-life of the acetyl group and the persistence of histone acetylation at steady state has suggested that acetylation is highly dynamic. As the histone acetylation and the deacetylation are both generated by specific enzymes, this led Loidl and colleagues to propose that the level of histone acetylation results from the antagonist activities of histone acetyltransferases and deacetylases [65]. It is only recently that the genome-wide mapping of HATs and HDACs revealed that the enzymes antagonistically controlling histone acetylation preferentially co-targeted active genes [66]. Hence, this provided indirect evidence that histone acetylation is a highly dynamic process within the living cell, rather than a simple persisting hallmark used as a platform, and allowed for the discrimination of actively transcribed chromatin from the rest of the genome. However, while these experiments clearly demonstrated that HATs and HDACs are in vicinity of active chromatin, they did not clarify whether both activities act on nucleosomal histones.

## 8. Histone Acetylation Coordinates Chromatin Dynamics

**Figure 1:**
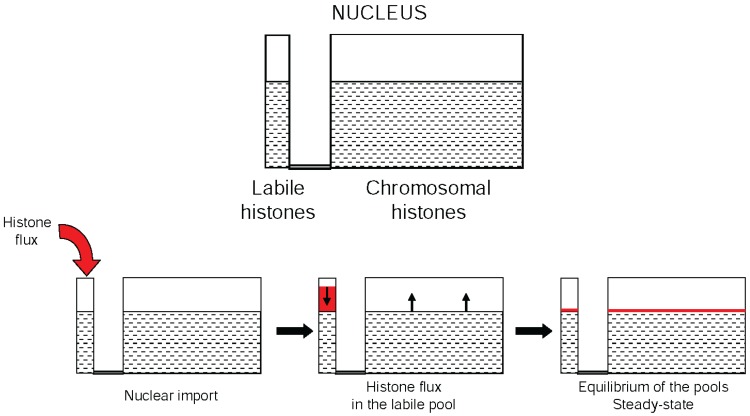
Schematic representation of the histone pools in the nucleus. The top scheme represents nuclear histones compartmentalized into two reservoirs, the labile histones (associated with various chaperones) and the chromosomal histones. The lower schemes illustrate the impact of the arrival of histones within the nucleus on the equilibrium of the histones’ reservoirs.

Transcription has been the focus of extensive studies that have allowed a precise description of features correlating with the transcription of the DNA double helix into RNA molecules in a chromatin context. Here, we have stated the major events accounting for this chromatin activity. However, it is only recently that we have provided a comprehensive model of the orchestration of the different highly dynamic processes that coordinate transcription [67]. *In vitro* and *in vivo* experiments have demonstrated that transcription implies chromatin dynamics, which can be achieved by the sliding of the histone octamer and histone exchange, or stochastic histone displacement. In any case, following transcription chromatin structure has to be regained efficiently. To investigate effective histone mobility, we have shown that 10%–15% of the nuclear histones are labile upon treatment with low concentration of Triton, in synchronous macroplasmodia of *Physarum* harvested in G2-phase. Interestingly, the analyses of the labile fraction of nuclear histones showed that a significant amount of H4 was acetylated. That the presence of non-chromatinized histones is conserved is suggested by the presence of different histone chaperones that have been isolated in various models, and that are necessary to prevent toxic effects of histones [68,69]. A unique feature of the model system *Physarum* is the capacity to spontaneously internalize exogenous proteins. Thus, it is possible to incorporate defined amounts of exogenous histones in S-phase and to examine their fate throughout the cell cycle. The titration of the exogenous H3/H4 exhibited two distinct distributions in early G2-phase, right after their incorporation in S-phase. At lower concentrations, exogenous histones were assembled into chromatin, while at higher amounts exogenous histones were present both in chromatin and in a labile nuclear pool. Even though the exogenous histones were incorporated in trace amounts (<1%–2%), clearly these results showed that the amounts of histones influenced their distribution in nuclei. It is uncertain that all eukaryotes present the same feature as *Physarum* with regard to the amount of histones, although the evidence of histone chaperones in a number of model systems suggests the existence in nuclei of a labile pool of non-chromosomal histones. The data in *Physarum* showed the existence in nuclei of a fragile equilibrium between the chromosomal pool of histones and the labile pool, which over time reached a steady state. Notably, the two pools of histones exhibited stringent size differences, as the chromosomal pool corresponded to ~85% of the nuclear histones, and the labile pool to ~15%. Obviously, any requirement in chromosomal histones, even small, will induce significant changes in the labile pool, as the histones first need to be transported into nuclei prior to being assembled into chromatin. Thus, we propose that the nuclear histones are composed of two connected reservoirs corresponding to the chromosomal histones and the labile histones, which are constantly equilibrated (Figure 1). Consistent with the biochemical investigations of Studitsky and collaborators [42,70], the analyses of core histone dynamics at the steady-state exhibited a low rate of exchange of the H3/H4 tetramer and a greater rate of exchange of H2A/H2B dimers, in correlation with the passage of RNA polymerase II. However, similar to the experiments in *Drosophila* [45], the ribosomal RNA genes presented a rapid eviction of the exogenous tetramer, showing a turnover of nucleosomes at this specific region of the genome that might be due to the velocity of transcription [44,48]. Nonetheless, the studies of the steady state did not provide a complete view of the actual dynamics of the histone. Thus, experiments to determine the rate of nucleosome exchange were carried out before the histone tracer reached the equilibrium in the two pools. These revealed that the nucleosome dynamics were directly correlated with the amount of exogenous histones [67]. Clearly, these experiments with the *Physarum* model system showed that nuclear histones are partitioned into two pools (chromosomal pool and labile free pool) that are in a fragile equilibrium that can be disturbed even with trace amounts of supplementary histones. Whether this feature is relevant for other organisms or cell types remains elusive, although FRET experiments on core histones in human cells have revealed that a small fraction of H3 is mobile [71].

**Figure 2:**
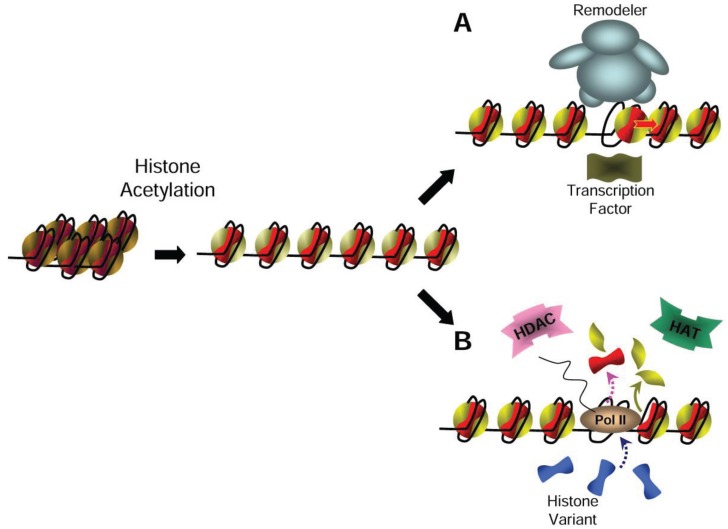
Model of the effects of histone acetylation on chromatin structure and dynamics. The acetylation of histones induced a decrease in nucleosome stacking involving chromatin remodeling at the promoter level (**A**) and within the coding region (**B**). Notably, the relaxation of chromatin in (**A**) can be promoted by HAT activity associated with transcription factors. In (**B**) the dashed arrows correspond to stochastic events such as octamer exchange, in contrast to the exchange of the H2A/H2B histone dimer, which is mechanistically linked to RNA polymerase II passage through the nucleosome (green arrow).

Using this original and unique experimental approach of incorporation of exogenous histones into *Physarum* macroplasmodia, we have also examined the dynamics of acetylated histones within nucleosomes. Experiments carried out with genetic mimics of acetylated H4 exhibited striking differences in nucleosome dynamics relative to the exchange of wild type exogenous histones. Indeed, the data revealed that the neutralization of lysine charge of H4 promotes the eviction of the nucleosome within the coding region when associated with euchromatin. Although it has been shown that the distinct positions of the acetyl group within H4 affected H4 partitioning within the two nuclear pools, experiments on exogenous histone incorporation did not demonstrate that specific acetylation contributes to the disassembly of nucleosome. However, it can be proposed that the greater displacement of the acetylated core histone facilitates the progression of the RNA polymerase on the DNA molecule that is no longer occluded by histone–DNA contacts. In addition, the fact that histone eviction was not observed at the steady state implies that displaced histones are rapidly replaced by available histones of the liable pool to regain chromatin structure. This model involving the displacement of acetylated histones from chromatin is quite attractive as it perfectly accommodates the different reported features of transcribed genes, including the co-localization of the antagonist enzymes HAT/HDAC [66], the enrichment in H3.3 of active chromatin [45,46,47], the short half-life of acetyl group within the histone lysines [64], and the platform mechanism of the acetyl group increasing the local concentration in bromodomain-containing factors [38,41] (Figure 2).

## 9. Perspectives

Over the past decades, many research groups have focused on the identification and characterization of novel histone modifications. To date, hundreds of individual modifications and their combination have been reported and their links with chromatin activities have been demonstrated. This large section of the literature has provided the basis for modern epigenetics. However, how all these chromatin hallmarks affect the dynamics at the nucleosomal level is still poorly understood. To gain insights into the role of the epigenetic modifications, it would be important to focus on the actual dynamics of the marks and their persistence in chromatin, rather than accumulating snapshots in distinct experimental conditions that are often difficult to bring together. The data supporting the dynamics of the histone acetylation have provided new insights into the role of this modification in transcription. It has been well demonstrated that acetylation is not the only histone modification involved in transcription. For instance, the methylation of H3 lysine 4 is often taken as model histone modification for active chromatin. Nonetheless, only few studies have examined the dynamics of this modification and the links with acetylation. Importantly, though the present review has focused on histone tail acetylation, recent works have also identified modifications within the histone globular domains. The analyses of these latest posttranslational modifications have revealed that they affect nucleosome stability and transcription [72,73,74]. Even though the link between histone acetylation and transcription was discovered a half century ago, during the past years a particular interest has emerged in the development of inhibitors targeting the dynamics of histone acetylation, which represents a promising avenue for cancer therapy.
